# Regional Diversity of Maritime Antarctic Soil Fungi and Predicted Responses of Guilds and Growth Forms to Climate Change

**DOI:** 10.3389/fmicb.2020.615659

**Published:** 2021-01-26

**Authors:** Kevin K. Newsham, Marie L. Davey, David W. Hopkins, Paul G. Dennis

**Affiliations:** ^1^ British Antarctic Survey, Natural Environment Research Council, Cambridge, United Kingdom; ^2^ Norwegian Institute for Nature Research, Trondheim, Norway; ^3^ Scotland’s Rural College, Edinburgh, United Kingdom; ^4^ School of Earth and Environmental Sciences, The University of Queensland, Brisbane, QLD, Australia

**Keywords:** Agaricales, ascomycetes, climate warming, phylogenetic marker (ITS2) sequencing, lichenised fungi, maritime Antarctica, saprotrophic fungi, yeasts

## Abstract

We report a metabarcoding study documenting the fungal taxa in 29 barren fellfield soils sampled from along a 1,650 km transect encompassing almost the entire maritime Antarctic (60–72°S) and the environmental factors structuring the richness, relative abundance, and taxonomic composition of three guilds and growth forms. The richness of the lichenised fungal guild, which accounted for 19% of the total fungal community, was positively associated with mean annual surface air temperature (MASAT), with an increase of 1.7 operational taxonomic units (OTUs) of lichenised fungi per degree Celsius rise in air temperature. Soil Mn concentration, MASAT, C:N ratio, and pH value determined the taxonomic composition of the lichenised guild, and the relative abundance of the guild was best predicted by soil Mn concentration. There was a 3% decrease in the relative abundance of the saprotrophic fungal guild in the total community for each degree Celsius rise in air temperature, and the OTU richness of the guild, which accounted for 39% of the community, was negatively associated with Mn concentration. The taxonomic composition of the saprotrophic guild varied with MASAT, pH value, and Mn, NH_4_^+^-N, and SO_4_^2−^ concentrations. The richness of the yeast community, which comprised 3% of the total fungal community, was positively associated with soil K concentration, with its composition being determined by C:N ratio. In contrast with a similar study in the Arctic, the relative abundance and richness of lichenised fungi declined between 60°S and 69°S, with those of saprotrophic Agaricales also declining sharply in soils beyond 63°S. Basidiomycota, which accounted for 4% of reads, were much less frequent than in vegetated soils at lower latitudes, with the Ascomycota (70% of reads) being the dominant phylum. We conclude that the richness, relative abundance, and taxonomic composition of guilds and growth forms of maritime Antarctic soil fungi are influenced by air temperature and edaphic factors, with implications for the soils of the region as its climate changes during the 21st century.

## Introduction

Although recent studies have described the fungi present in continental Antarctic soils and identified the factors controlling their activities and frequencies (Chan et al., 2013; Dreesens et al., 2014; Archer et al., 2019; Canini et al., 2020; Sannino et al., 2020), our understanding of the fungi inhabiting maritime Antarctic soils is still in its infancy. This knowledge gap is significant, since fungi have pivotal roles in all terrestrial ecosystems as decomposers of organic matter and as partners in symbioses, and notably the lichen symbiosis, which is widespread in maritime Antarctica (Øvstedal and Smith, 2001). A further imperative to study soil fungi in maritime Antarctica is that the region underwent significant change in the latter half of the 20th century, with near surface air temperatures rising by up to 3°C between the 1950s and late 1990s (Adams et al., 2009). Although this warming trend has recently slowed (Turner et al., 2016), surface air temperatures in the region are predicted to increase during the 21st century as greenhouse gases accumulate in the atmosphere (Bracegirdle et al., 2008; Bracegirdle and Stephenson, 2012). Given the roles of fungi in the decomposition process and as lichen symbionts, it follows that changes to their diversity caused by rising temperatures could influence the responses of maritime Antarctic terrestrial ecosystems to climate change.

The responses of soil fungal communities to the steep changes in environmental conditions across the maritime Antarctic – notably the significant reductions in surface air temperatures at higher latitudes in the region – have been foci for several studies (Lawley et al., 2004; Yergeau et al., 2007a; Dennis et al., 2012; Newsham et al., 2016). Although edaphic factors such as soil pH, Mn concentration, and C:N ratio account for variation in community composition (Yergeau et al., 2007a; Dennis et al., 2012; Newsham et al., 2016), the species richness of all soil fungi in the region is primarily determined by mean annual surface air temperature (MASAT), with reductions in the total number of fungal species in colder soils at higher latitudes (Newsham et al., 2016). However, the environmental factors controlling the relative abundances and taxonomic compositions of the predominant guilds and growth forms of fungi present in maritime Antarctic soils have yet to be identified. In the Arctic, research along a transect from Alaska (69°N) through to Ellef Ringnes Island in the High Arctic (79°N) indicates significant responses of three soil fungal guilds and growth forms to changes in environmental conditions at higher latitudes, with significant increases in the relative abundances of lichenised fungi and yeasts, and reductions in those of the ectomycorrhiza-forming fungi, in more northerly soils (Timling et al., 2014). It is possible that the abundances of lichen-forming fungi and yeasts might similarly alter across the maritime Antarctic, but studies have hitherto not addressed this possibility. In particular, increases in the abundances of yeasts might be anticipated in soils at higher latitudes in the region, since these are apparently the only fungi that can be isolated from soils in the McMurdo Dry Valleys in the continental Antarctic (Atlas et al., 1978), considered to be among some of the most hostile environments for life on Earth. Ectomycorrhizal symbioses, which are formed predominantly by basidiomycete fungi (Smith and Read, 2008), appear to be entirely absent from Antarctica owing to a lack of woody host plant species (Newsham et al., 2009). However, field observations suggest that another group of basidiomycetes, the sporocarp-forming Agarics, might decline in abundance at higher latitudes, with fruiting bodies of these fungi typically being restricted to habitats at the lowest latitudes in maritime Antarctica (Pegler et al., 1980).

Next generation sequencing, which offers greater sampling depth than cloning methods (Lawley et al., 2004; Yergeau et al., 2007a; Bridge and Newsham, 2009), has previously been used to determine the taxonomic compositions of soil fungal communities in the Americas, Asia, Africa, Europe, and Australasia (Tedersoo et al., 2014; Bahram et al., 2018; Egidi et al., 2019). However, Antarctica has been neglected in these studies, and, although this technique has been used several times previously to characterise the fungal communities of maritime Antarctic soils, detailed taxonomic information for the fungi present in a wide range of soils has yet to be presented. For example, two such studies have reported fungal taxa in two vegetated soils sampled from the region (Cox et al., 2016, 2019). Another study, despite basing its analyses on all fungal taxa in 29 soils sampled from across almost the entire maritime Antarctic, reported only the 50 most frequent taxa (Newsham et al., 2016). Here, we use the data reported by this study to characterise in much greater detail the regional diversity of maritime Antarctic soil fungi and to investigate the environmental factors structuring the compositions of lichenised and saprotrophic fungal guilds and yeast communities in the region, which were not addressed by Newsham et al. (2016). We sought to identify how environmental factors that can be expected to change over future decades in maritime Antarctica, such as MASAT, soil C:N ratio, and N concentrations (Newsham et al., 2016; Hill et al., 2019), are associated with the species richness, abundance, and composition of each of these guilds and growth forms, in order to predict how they will respond to further climate change in the region.

## Materials and Methods

**Figure 1:**
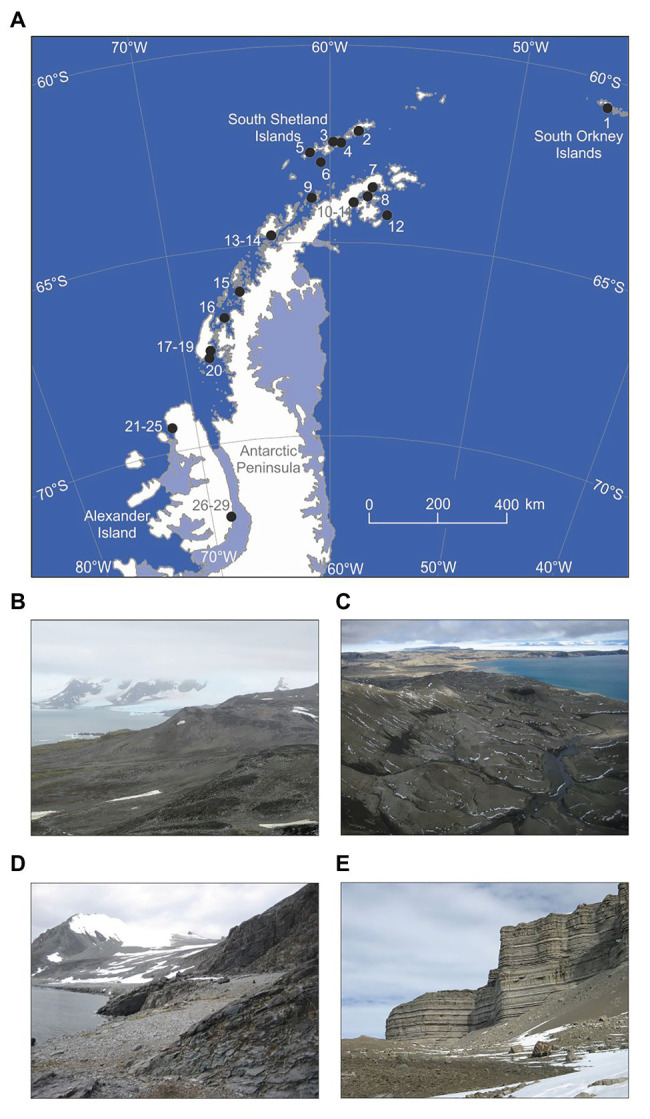
**(A)** Locations of sampling sites. 1: Wynn Knolls, Signy Island (60.701°S, 45.661°W), 2: Keller Peninsula, King George Island (62.086°S, 58.399°W), 3: Orión Point, Greenwich Island (62.447°S, 59.736°W), 4: Edwards Point, Robert Island (62.460°S, 59.509°W), 5: South Beaches, Byers Peninsula, Livingston Island (62.655°S, 61.090°W), 6: Whalers Bay, Deception Island (62.977°S, 60.552°W), 7: Newsham Nunatak (63.559°S, 57.823°W), 8: Cape Lachman, James Ross Island (63.782°S, 57.783°W), 9: Spert Island (63.844°S, 60.945°W), 10–11: Alectoria Island (63.977°S, 58.640°W), 12: Marambio Station (ARG), Seymour Island (64.236°S, 56.626°W), 13: Port Lockroy, Goudier Island (64.817°S, 63.483°W), 14: Yelcho Station (CHL), Wiencke Island (64.894°S, 63.553°W), 15: Cape Evenson, Antarctic Peninsula (66.145°S, 65.717°W), 16: Detaille Island (66.790°S, 66.869°W), 17–19: Blaiklock Island, Rothera Point and Lagoon Island (67.543°S, 67.198°W, 67.568°S, 68.114°W and 67.594°S, 68.247°W, respectively), 20: Jenny Island (67.731°S, 68.365°W), 21–25: Hopkins Ridge and Mount Holt, Alexander Island (69.366°S, 71.842°W; 69.366°S, 71.844°W; 69.367°S, 71.844°W; 69.368°S, 71.844°W and 69.408°S, 71.665°W), and 26–29: Mars Oasis, Alexander Island (71.878°S, 68.248°W). **(B–E)** Show images of sites 1, 12, 20, and 26–29, respectively.

Soils were sampled as described by Newsham et al. (2016) in November 2007 to February 2008 from 29 sites along a 1,650 km latitudinal transect between Signy Island in the South Orkney Islands (60°S) and south-eastern Alexander Island (72°S; Figure 1A). The soils that were collected were devoid of vegetation (Figures 1B–E) and were hence representative of the barren soils that typically form in maritime Antarctica. The sampling sites were either accessed by small boat or helicopter deployed from a ship, by fixed wing aircraft fitted with skis, or by foot from research stations. At each site, five samples of soil (0–50 mm depth, *c*. 50 cm^3^) were placed into DNA/RNAase-treated tubes and bulked. Biological soil crusts, stones, and visible lichen thalli were avoided. The soils were frozen immediately at *c*. −80°C in a mixture of dry ice and ethanol and were kept at this temperature until processing.

Following the methods described by Newsham et al. (2016), 20 edaphic factors were measured in each 4-mm sieved soil, *viz*., moisture concentration, pH (H_2_O), electrical conductivity, the concentrations of total organic C and N, Ca, Cu, Fe, K, Mg, Mn, Ni, P, and Zn, and those of water-extractable PO_4_^3−^, SO_4_^2−^, Cl^−^, NH_4_^+^-N, NO_3_^−^-N/NO_2_^—^N, and dissolved organic carbon (DOC). Soil C:N ratio was also calculated. Data for each of the 29 soils are shown by Dennis et al. (2020). The soils that were sampled were on average slightly acidic, had low total organic C and N concentrations, and, in comparison with mineral soils at lower latitudes, had low NH_4_^+^-N, NO_3_^−^-N/NO_2_^—^N, and K concentrations, low to moderate concentrations of PO_4_^3−^, and high concentrations of K, Ca, Mg, and Mn (cf. Allen, 1989; Table 1). The remote locations of most of the studied sites precluded the direct measurement of soil temperatures, and so MASAT at each location for 2007 was derived from the Regional Atmospheric Climate Model over Antarctica (Van Lipzig et al., 1999), gridded at a horizontal resolution of 55 × 55 km. Regression analyses indicated a significant decline in MASAT from −4 to −11°C, and an increase in total C:N ratio from 3 to 10, between 60°S and 72°S (both *r*^2^ = 31–33%, *p*<0.002), but no significant changes in other edaphic factors or altitude (Newsham et al., 2016).

**Table 1:** Summary edaphic factors of the sampled soils.

Parameter	Mean	Range
Moisture concentration	19.31	3.88–71.03
pH (H_2_O)	6.64	5.26–7.76
Electrical conductivity	55.54	14.45–415.80
Organic C concentration	1.38	0.01–16.76
N concentration	0.21	0.01–2.57
Organic C:N ratio	6.64	1.59–13.87
Ca concentration	28.67	2.37–244.18
Cu concentration	66.10	0.00–290.40
Fe concentration	44.50	3.99–105.43
K concentration	2.43	0.11–10.00
Mg concentration	11.26	2.29–60.66
Mn concentration	1.06	0.15–3.67
Ni concentration	22.49	4.00–153.33
P concentration	5.41	0.16–58.84
Zn concentration	95.46	13.00–460.67
Dissolved organic C concentration	1.65	<0.01–11.45
Dissolved NO_3_^−^-N/NO_2_^−^-N concentration	0.08	<0.01–0.96
Dissolved NH_4_^+^-N concentration	0.55	<0.01–1.61
Dissolved SO_4_^2−^ concentration	11.52	<0.01–180.49
Dissolved PO_4_^3−^-P concentration	4.48	<0.01–63.39
Dissolved Cl^−^ concentration	33.03	3.40–278.15

Units are mg kg^−1^ dry soil for all parameters except concentrations of moisture (%), Mg, P, K, Ca, Mn, and Fe (g kg^−1^), electrical conductivity (μS cm^−1^), pH, and C:N ratio (dimensionless). For methods, see Newsham et al. (2016).

The methods used to extract DNA from soil and amplify internal transcribed spacer 2 (ITS2) encoding genes, and the downstream processing of sequence data (which have been deposited in the NCBI short read archive), are described in detail by Newsham et al. (2016). Briefly, total DNA was extracted under aseptic conditions from soil using a commercial soil DNA isolation kit. The ITS2 region of fungal ribosomal RNA encoding genes was amplified by PCR using the primers gITS7 (5' GTGARTCATCGARTCTTTG; Ihrmark et al., 2012) and ITS4 (5' TCCTCCGCTTATTGATATGC; White et al., 1990). The use of these primers avoided the distortion of community composition associated with the ITS1F/ITS4 primer pair in next generation sequencing studies (Ihrmark et al., 2012). The forward primer was 5'-labelled with the 454 FLX sequencing primer adapter B sequence and the reverse primer with a 5'-labelled sample specific barcode sequence and the 454 FLX sequencing primer adapter A sequence. Amplicons from PCRs were purified and quantified before each sample was pooled, purified again, and then 454 pyrosequenced (Margulies et al., 2005) at a commercial facility. The sequences were quality filtered and dereplicated using the QIIME script split_libraries.py procedure, with the homopolymer filter deactivated (Caporaso et al., 2010). Acacia v. 1.48 (Bragg et al., 2012) was used to correct homopolymer errors and fungal ITS2 sequences were extracted with ITSx v. 1.0.9 (Bengtsson-Palme et al., 2013). Sequences were checked for chimeras against ITS2 sequences in UNITE v. 8.2 (Nilsson et al., 2015) using UCHIME v. 3.0.617 (Edgar, 2010). operational taxonomic units (OTUs) that could not be assigned to the kingdom Fungi were deleted from the dataset. At least 1,435 non-chimeric quality-filtered ITS2 sequences were derived from each soil sample. The sequences were clustered using UCLUST v. 1.2.22, at 97% similarity. UNITE v. 8.2 taxonomy (Kõljalg et al., 2013) was assigned to representative OTU sequences using BLAST v. 2.2.30, with the exception that members of *Mortierella* were assigned to the Mucoromycotina (Hibbett et al., 2007). Tables containing the abundances of different OTUs and their taxonomic assignments in each sample were generated and the number of reads was rarefied to 1,400 per sample. Richness was defined as the total number of observed OTUs per sample. In addition, guilds and growth forms were assigned to each OTU using FUNguild v. 1.0 (Nguyen et al., 2016), including taxa for which the trophic mode assignments were “highly probable” or “probable,” and with minor manual adjustments (Supplementary Table S1). This procedure assigned a guild or growth form to 198, 238, and 66 OTUs of lichen-forming fungi, saprotrophic fungi and yeasts, respectively (Supplementary Table S1). Saprotrophic fungi included both filamentous fungal and obligate and facultative yeast genera (Supplementary Table S1). Only 10% of OTUs could be assigned to mycorrhizal, mycoparasitic, plant parasitic, or animal pathogenic fungal guilds, and so were not included in further guild level analyses.

Data analyses were implemented using R v. 3.6.3. Graphic summaries of the relative abundances of members of the soil fungal community were generated using the metacoder package (Foster et al., 2017). Regression analyses were used to determine associations between latitude and the number of OTUs and relative abundances in the total fungal community of each guild or growth form. Owing to there being *a priori* evidence of reduced frequencies of basidiocarp-forming Agarics at higher latitudes in maritime Antarctica (Pegler et al., 1980), regression analyses were also used to determine the association between the relative abundances of the saprotrophic Agaricales and latitude. Relationships between MASAT and edaphic factors and the richness and relative abundances of OTUs assigned to lichenised and saprotrophic fungal guilds, or to yeasts, were assessed using multiple linear regression with forward selection of significant predictors. The associations between MASAT and edaphic factors and the compositions of lichenised and saprotrophic fungal guilds, or yeast communities, were assessed using permutational multivariate analysis of variance (PERMANOVA), as implemented in the vegan package (Oksanen et al., 2017). The relative abundances of OTUs were Hellinger transformed prior to analysis and parsimonious PERMANOVA models were built by forward selection of significant predictors.

## Results

### Taxonomic Composition of Maritime Antarctic Soil Fungal Communities

After rarefying the sequencing data to 1,400 reads per sample, a total of 1,220 fungal OTUs were recorded in the 29 soils, with 770 OTUs being assigned to taxa (Supplementary Table S1). Ascomycota dominated the soil fungal community, with 69.89% of reads being assigned to this phylum, whereas the phylum Basidiomycota accounted for only 4.36% of reads (Supplementary Table S1). Of the basal fungi, reads assigned to the Chytridiomycota, Glomeromycota and Mucoromycotina were present at relative abundances of 0.002, 0.007, and 8.863%, respectively (Supplementary Table S1). Other basal phyla or sub-phyla were not recorded in the soils.

**Figure 2:**
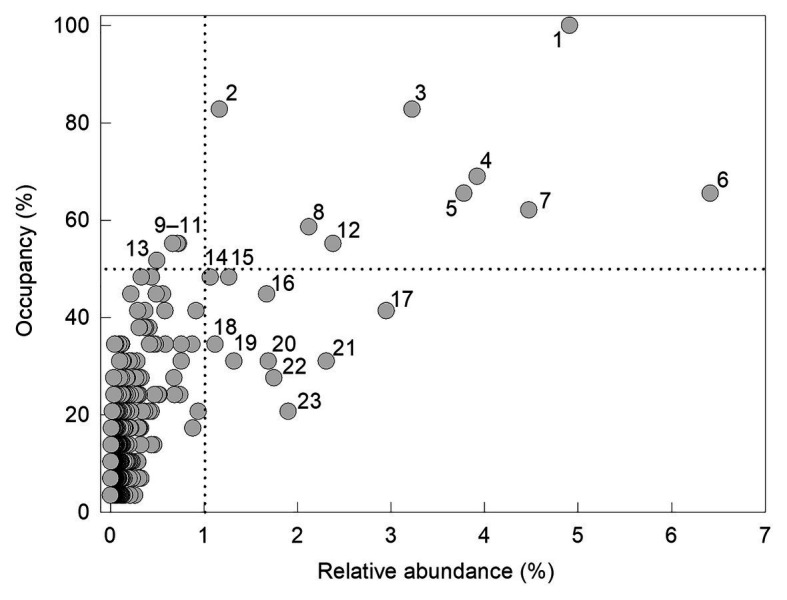
Occupancy of operational taxonomic units (OTUs) as a function of their relative abundance. OTUs recorded in >50% of soils and at relative abundances of >1% (denoted by the dotted lines) are labelled (1: *Pseudogymnoascus roseus*, 2: *Cladosporium halotolerans*, 3: *Mortierella* sp., 4: unclassified ascomycete, 5: *Antarctomyces* sp., 6: *Fusarium* sp., 7: *Pseudeurotium hygrophilum*, 8: unclassified ascomycete, 9: *Atla* sp., 10: Verrucariaceae sp., 11: *Lobaria pseudopulmonaria*, 12: unclassified ascomycete, 13: *Catenulifera* sp., 14–15: unclassified ascomycetes, 16: *Lecidea cancriformis*, 17: *Mortierella polygonia*, 18–19: unclassified ascomycetes, 20: *Penicillium polonicum*, 21–22: *Verrucaria* spp., and 23: unclassified ascomycete).

There was a significant positive association between the occupancy of an individual OTU (the percentage of the 29 soils in which it was recorded) and its relative abundance (*r*^2^ adj. = 52.2%, *F*_1,1,218_ = 1332.7, *p*<0.001; Figure 2). Fungi with high occupancy or frequency, defined here as occurring in >50% of the soils and at >1% relative abundances, were almost exclusively members of the Ascomycota. The only OTU recorded in all 29 soils was *Pseudogymnoascus roseus* (Figure 2). The next most widespread OTUs, recorded in 24 (83%) of the soils, were a *Mortierella* species and *Cladosporium halotolerans*. Representatives of *Antarctomyces*, *Fusarium*, *Pseudeurotium*, and unclassified ascomycetes occurred in 18–20 (62–69%) of the soils. Members of the genera *Atla*, *Lobaria*, and *Catenulifera*, the family Verrucariaceae, along with two unclassified ascomycetes, occurred in 15–17 (52–59%) of the soils along the transect, but all of these taxa, except for the unidentified ascomycetes, occurred at relative abundances of <1% (Figure 2). Other taxa that occurred in <50% of the soils, but were present at relative abundances of >1%, were *Lecidea cancriformis*, *Mortierella polygonia*, *Penicillium polonicum*, *Verrucaria* spp., and unclassified ascomycetes (Figure 2).

### Taxonomic Composition of Guilds and Growth Forms

**Figure 3:**
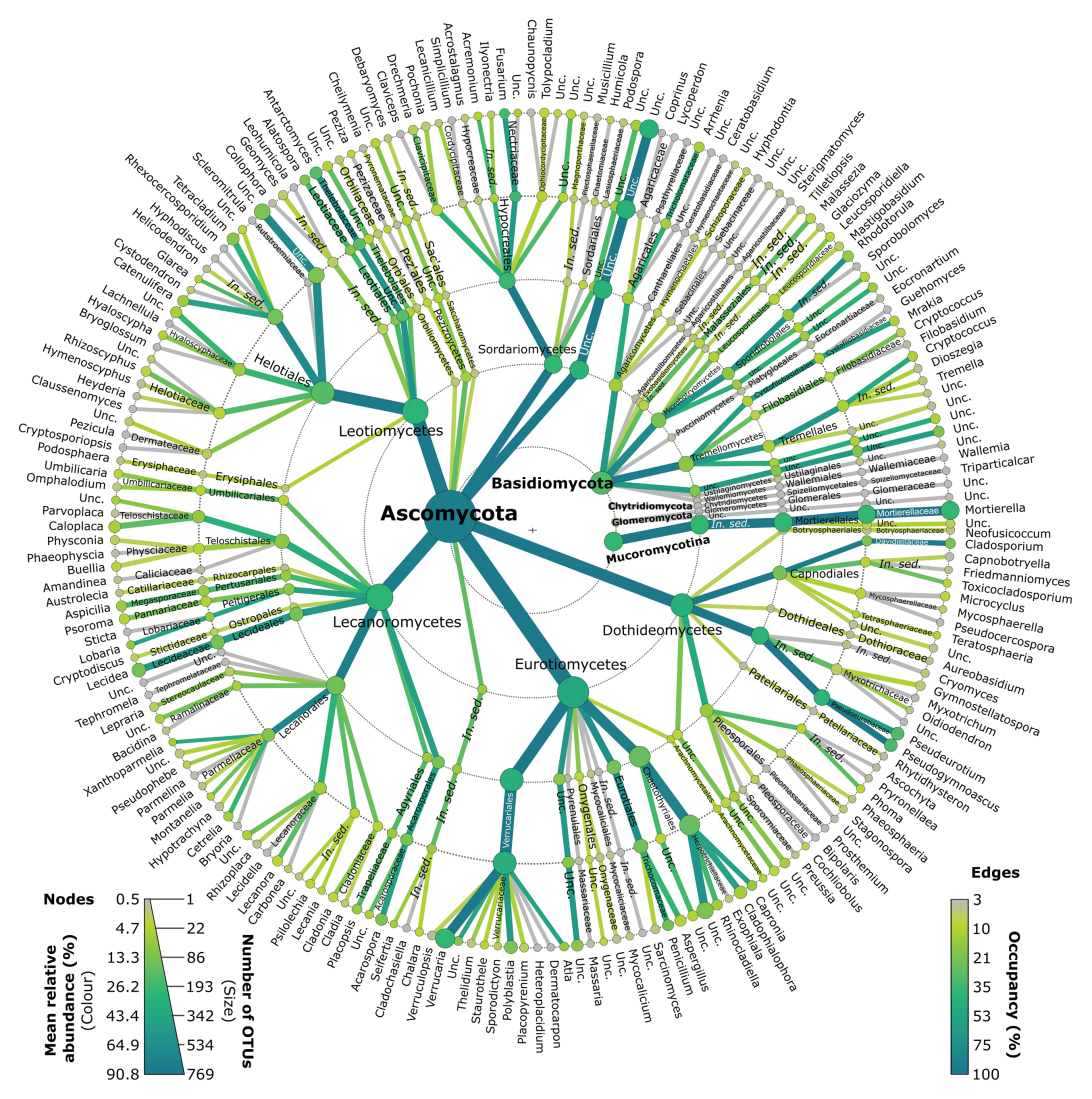
Metacoder representation of the taxonomic composition of the Antarctic fellfield soil fungal community. Nodes and branches are coloured by the total number of reads assigned to them, and are sized by numbers of OTUs. Edge colours represent occupancy. Unc., unclassified.

Lichenised fungi accounted for 18.8% of all reads, and primarily belonged to the Verrucariaceae (Verrucariales, Eurotiomycetes; Figure 3; Supplementary Table S1). Representatives of this family consisted primarily of *Verrucaria* OTUs (8.5% of reads), but also *Polyblastia* (1.6%), *Atla* (0.7%), *Staurothele* (0.1%), and six other genera (each <0.1%; Figure 3; Supplementary Table S1). Lichenised Lecanoromycetes were also frequent, including the genera *Lecidea* (2.0%), *Acarospora* (1.5%), *Lobaria* (0.7%), *Aspicilia* (0.6%), *Placopsis* (0.5%), *Xanthoparmelia* (0.4%), *Cetrelia* (0.4%), *Caloplaca* (0.3%), *Montanelia* (0.2%), *Psoroma* (0.2%), *Umbilicaria*, *Lecanora*, *Psilolechia*, and *Cladonia* (each 0.1%), and 20 others (each <0.1%; Figure 3; Supplementary Table S1).

Saprotrophic fungi accounted for 38.7% of all reads. Among the saprotrophs, members of the ascomycete classes Eurotiomycetes, Leotiomycetes, Dothideomycetes, and Sordariomycetes were frequent, with *Fusarium* (6.4%), *Pseudogymnoascus* (5.1%), *Pseudeurotium* (4.7%), *Antarctomyces* (3.9%), *Penicillium* (1.8%), *Cladosporium* (1.6%), *Alatospora* (1.1%), *Tetracladium* (1.1%), *Catenulifera* (0.5%), *Cladophialophora* (0.4%), *Rhinocladiella* (0.4%), *Hyaloscypha* (0.2%), *Aspergillus* (0.2%), *Acremonium* (0.2%), *Glarea* (0.2%), and *Phoma* (0.1%) being the most abundant genera detected, along with 22 other saprotrophic ascomycete genera (each <0.1%; Figure 3; Supplementary Table S1). The most abundant saprotrophic genus was *Mortierella* (8.8%), the only genus recorded in the Mucoromycotina (Figure 3; Supplementary Table S1).

The yeasts, the majority of which were basidiomycetes, accounted for 3.1% of all reads. Tremellomycetes and Microbotryomycetes were the most frequent classes, and consisted primarily of the obligate yeasts *Rhodotorula* (0.6%), *Cryptococcus* (0.3%), *Mrakia* (0.2%), and *Leucosporidiella* (0.2%), along with seven other yeast genera (Figure 3; Supplementary Table S1). Facultative ascomycete yeasts in the Chaetothyriales (Eurotiomycetes) were also recorded, with *Capronia* (0.6%), *Cladophialophora* (0.4%), and *Rhinocladiella* (0.4%) being the most frequent genera recorded in this order (Figure 3; Supplementary Table S1).

### Changes in Soil Fungal Community Composition With Latitude

#### Lichenised Fungi

**Figure 4:**
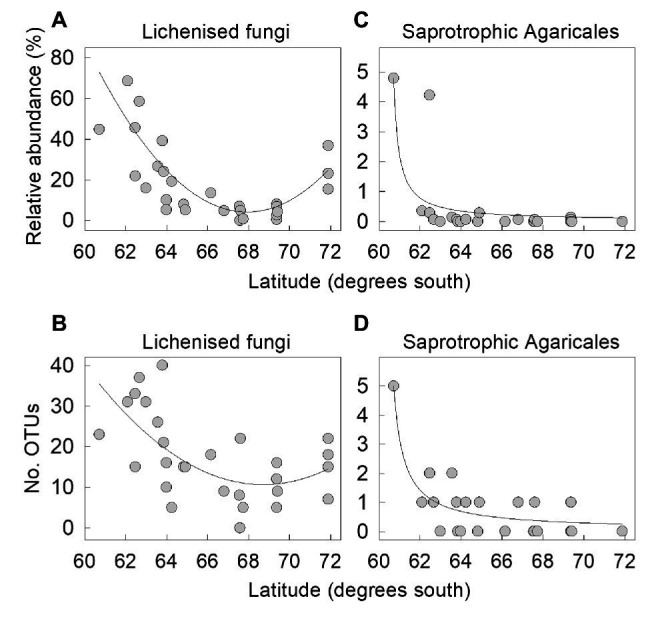
The relative abundances of **(A,C)** lichenised fungi and saprotrophic Agaricales as functions of latitude and the number of OTUs of **(B,D)** lichenised fungi and saprotrophic Agaricales as functions of latitude. Lines show significant first order polynomial **(A,B)** and hyperbolic **(C,D)** fits.

The proportion of lichenised fungi in the total soil fungal community changed significantly with latitude, with a sharp decline from 22 – 69% of the community in the South Orkney and South Shetland islands (60–63°S) to ≤8% on north-west Alexander Island at 69°S (Figure 4A). However, there was a subsequent increase in the relative abundance of lichenised fungi to 15–37% of the community on southern Alexander Island (72°S), resulting in a first order polynomial relationship between frequency and latitude (*r*^2^ adj. = 57.3%, *F*_1,28_ = 19.80, *p*<0.001). This increase was partly driven by increased abundance of *Acarospora*, *Polyblastia*, and *Cetrelia*, an observation supported by PERMANOVA analyses showing highly significant effects of latitude on the taxonomic composition of the lichenised fungal guild (*F*_1,27_ = 3.35, *p* = 0.001). The association between the OTU richness of lichenised fungi and latitude also followed a similar pattern, and was best described by a first order polynomial (*r*^2^ adj. = 34.8%, *F*_2,26_ = 8.48, *p*=0.001; Figure 4B).

#### Saprotrophic Fungi

Regression analyses indicated that neither the proportion of saprotrophs in the total fungal community (*r*^2^ adj. = 6.2%, *F*_1,27_ = 2.86, *p*>0.1) nor saprotrophic OTU richness (*r*^2^ adj. = 0.0%, *F*_1,27_ = 0.47, *p*>0.5) varied significantly with latitude (Supplementary Figure S1). However, regression analyses identified declining abundance of saprotrophic Agaricales in soils beyond 63°S, with a significant hyperbolic relationship between the relative abundance of the order and latitude (Figure 4C). These fungi, which were primarily *Arrhenia* OTUs (Supplementary Table S1), were relatively abundant (4.2–4.8%) in soils at the northern end of the transect sampled from Signy Island and Greenwich Island (60°S and 62°S, respectively) but did not exceed 0.4% abundance in the 27 other soils (*r*^2^ adj. = 58.9%, *F*_1,28_ = 31.85, *p*<0.001; Figure 4C). Saprotrophic Agaricales OTU richness followed a similar hyperbolic association, with five *Arrhenia* OTUs recorded in soil from Signy Island and 0–2 OTUs of saprotrophic Agaricales recorded in other, more southerly soils (*r*^2^ adj. = 70.9%, *F*_1,28_ = 23.18, *p*<0.001; Figure 4D).

#### Yeasts

The relative abundance of yeasts in the total fungal community, and the OTU richness of the growth form, did not vary with latitude (both *r*^2^ adj. = 0.0%, *F*_1,27_ = 0.07–0.17, both *p*>0.7; Supplementary Figure S1).

### Factors Structuring Guilds and Growth Forms

#### Lichenised Fungi

**Figure 5:**
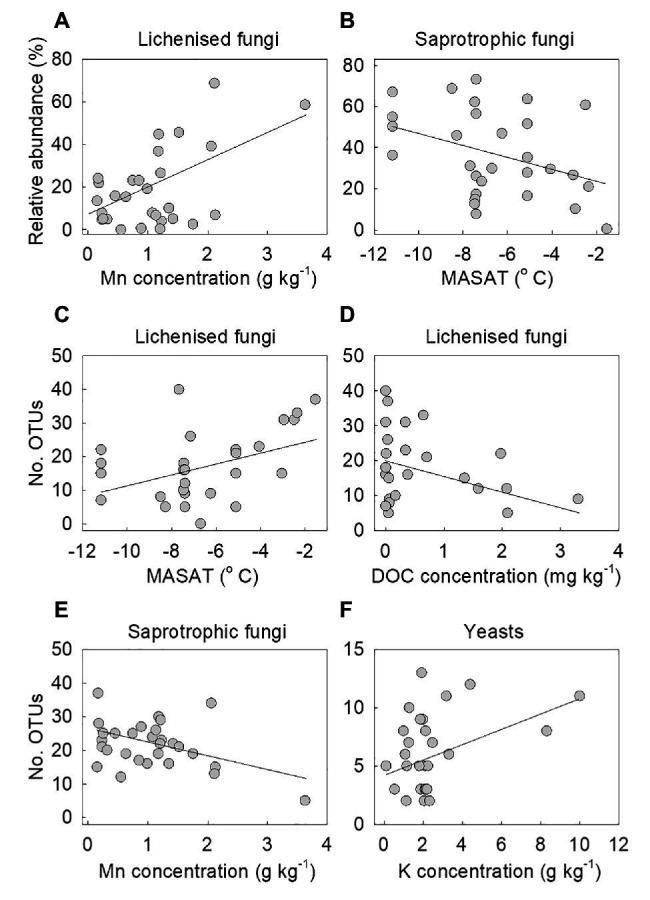
The relative abundances of **(A)** lichenised and **(B)** saprotrophic fungi as functions of soil Mn concentration and mean annual surface air temperature (MASAT), respectively, and the number of OTUs of lichenised fungi as a function of **(C)** MASAT and **(D)** dissolved organic carbon (DOC) concentration, and of **(E)** saprotrophic fungi as a function of Mn concentration and **(F)** yeasts as a function of K concentration. Lines are significant linear fits.

Multiple regression models using forward selection indicated that the relative abundance of lichenised fungi in the total fungal community was best predicted by soil Mn concentration, with a positive association between the abundance of the guild and this variable (*t* = 3.25, *p* = 0.003; Figure 5A). The OTU richness of the lichenised guild was best explained by MASAT, with soil DOC concentration and C:N ratio also predicting the numbers of OTUs of these fungi (Table 2). Lichenised fungal OTU richness was positively associated with MASAT (slope = 1.69 species per degree Celsius) and negatively so with DOC concentration (Figures 5C,D). PERMANOVA indicated that soil Mn concentration best predicted the composition of the lichenised fungal guild, with MASAT, C:N ratio, and pH value also predicting guild composition (Table 3).

**Table 2:** Data from multiple regression models using forward selection showing the final model best explaining observed OTU richness within guilds and growth forms of maritime Antarctic soil fungi.

Guild or growth form	Predictors	*t* value	*p*
Lichenised fungi	MASAT	4.87	<0.001
	Dissolved organic C concentration	−3.26	0.001
	C:N ratio	3.34	0.003
Saprotrophic fungi	Mn concentration	−2.31	0.029
Yeasts	K concentration	2.19	0.037

**Table 3:** Data from permutational multivariate analysis of variance (PERMANOVA) models using forward selection on Hellinger-transformed data showing the factors best explaining the taxonomic composition of guilds and growth forms of maritime Antarctic soil fungi.

Guild or growth form	Predictors	*r*^2^	*F* value	*p*
Lichenised fungi	Mn concentration	12.2	4.36	<0.001
	MASAT	10.4	3.71	<0.001
	C:N ratio	5.2	1.85	0.035
	pH value	5.1	1.84	0.036
Saprotrophic fungi	Dissolved NH_4_^+^-N concentration	9.1	3.19	<0.001
	MASAT	7.9	2.78	0.004
	pH value	5.5	1.95	0.033
	Mn concentration	5.9	2.09	0.022
	Dissolved SO_4_^2−^ concentration	6.4	2.27	0.021
Yeasts	C:N ratio	8.8	2.61	0.005

#### Saprotrophic Fungi

The relative abundance of saprotrophic fungi was best predicted by MASAT, with a negative association between the abundance of the guild in the total fungal community and this variable (*t* = −2.19, *p* = 0.037; slope = −3.01% per degree Celsius; Figure 5B). The OTU richness of the guild was negatively associated with soil Mn concentration (Table 2; Figure 5E). Its composition was best predicted by dissolved NH_4_^+^-N concentration, with significant, but weaker, effects of MASAT, pH value, and Mn and dissolved SO_4_^2−^ concentrations (Table 3).

#### Yeasts

None of the measured environmental and edaphic factors significantly (*p*<0.05) predicted the relative abundance of the yeasts in the total fungal community. Multiple regression models indicated that yeast OTU richness was positively associated with soil K concentration (Table 2; Figure 5F), and PERMANOVA analyses showed that the taxonomic composition of the community was significantly associated with soil C:N ratio (Table 3).

## Discussion

Maritime Antarctic terrestrial ecosystems underwent significant changes during the second half of the 20th century, with rises in MASAT of 0.13–0.55°C per decade being recorded across the region (Adams et al., 2009). Although this warming trend has since slowed (Turner et al., 2016), temperature increases of 2–4°C are predicted in the region by the end of the 21st century as greenhouse gases continue to accumulate in the atmosphere (Bracegirdle et al., 2008; Bracegirdle and Stephenson, 2012). Building on a previous report predicting that the total species richness of maritime Antarctic soil fungi will increase with rising air temperatures (Newsham et al., 2016), the observations here indicate that increases in MASAT will differentially affect two guilds of fungi inhabiting the fellfield soils of the region, with the lichenised fungal guild likely to show increases in species richness, and the saprotrophic guild potentially showing decreases in its relative abundance, as the region warms. We anticipate that the significant influence of MASAT on the lichenised and saprotrophic fungal guilds reflect not only the positive association between air temperature and soil temperature (Qian et al., 2011; Fang et al., 2019), with consequent effects on soil fungal diversity (Newsham et al., 2016), but are also indicative of the influence of temperature on water availability. Air temperatures in maritime Antarctic terrestrial ecosystems are routinely close to freezing point during summer (Convey et al., 2018), and there is thus a strong positive effect of increasing temperature on the availability of liquid water. Although unlikely to explain the reduced relative abundance of saprotrophs, higher water availability, combined with more frequent rainfall in warmer habitats (Turner et al., 2002), would allow increased productivity and metabolism of lichenised fungi, and permit a switch from survival to growth and dispersal strategies, explaining the positive association between the species richness of the guild and MASAT (Green et al., 2011). Thus, although instantaneous measurements of soil moisture concentration were not a significant predictor in the analyses here, we anticipate that long-term measurements of water availability would have been associated with soil fungal diversity.

Although MASAT was a significant predictor for the richness, relative abundance, and composition of the lichenised and saprotrophic fungal guilds, seven edaphic factors (pH value, C:N ratio, and the concentrations of Mn, K, SO_4_^2−^, DOC, and NH_4_^+^-N) also correlated with the diversities of the fungal guilds and growth forms studied here. It is important to note that, owing to the correlative nature of the analyses reported here, we cannot be certain of causal relationships between some of the edaphic factors and soil fungal diversity. Notably, the reasons for the relationships between guild diversity and soil Mn concentration, which also partially predicts total soil fungal community composition (Newsham et al., 2016), are presently unclear, and changes to the concentrations of this element, along with those of K, SO_4_^2−^, and H^+^, seem unlikely in warmer and wetter maritime Antarctic soils. However, changes to soil C:N ratio and concentrations of DOC and NH_4_^+^-N are plausible as the climate of the region changes. The analyses here indicate that declines in the ratio of C:N in warmer and less arid soils (Callesen et al., 2007), which most probably arise from accelerated C cycling (Swift et al., 1979), will influence the compositions of the yeast community and the lichenised fungal guild, and will offset any increases in the species richness of lichenised fungi resulting from rising air temperatures. The analyses here also indicate that increases in soil DOC and NH_4_^+^-N concentrations, which can be anticipated as decomposition, and particularly that of amino acids and proteins, accelerates in warmer and less arid maritime Antarctic soils (Kalbitz et al., 2000; Bond-Lamberty and Thomson, 2010; Hill et al., 2011, 2019), will alter the composition of the saprotrophic fungal guild and, as for C:N ratio, offset the increasing species richness of lichenised fungi associated with increasing MASAT.

While previous analyses have indicated that rising air temperature is associated with increased abundances of three OTUs assigned to the Verrucariaceae, and a decrease in a further OTU in the family (Newsham et al., 2016), the data reported here suggest an increase of 1.7 species of all lichenised fungi per degree Celsius rise in MASAT. These were typically mycobionts of saxicolous lichens, such as *Verrucaria*, *Polyblastia*, *Lecidea*, and *Acarospora* (Øvstedal and Smith, 2001). Other studies have also found higher numbers of lichen species in warmer habitats along the Antarctic Peninsula, albeit at higher rates of change, with increases of 24 usually saxicolous species per degree Celsius rise in MASAT (Peat et al., 2007; Green et al., 2011). Given the roles of the lichen symbiosis in cold and arid environments – such as the fixation of C and N from the atmosphere, mineral weathering, and the stabilisation of biological soil crusts (Gold and Bliss, 1995; Pointing and Belnap, 2012; Colesie et al., 2014) – it seems likely that increased species richness of lichenised fungi will have positive impacts on Antarctic terrestrial ecosystems. However, it is important to note that the studies of Peat et al. (2007) and Green et al. (2011) analyzed records of lichen specimens lodged in herbaria, whereas the study here was based on DNA amplified from soils from which visible lichen thalli were absent, suggesting that maritime Antarctic fellfield soils are rich sources of inoculum for the mycobionts of the saxicolous lichens that are frequent in the region (Øvstedal and Smith, 2001).

Saprotrophic fungi are central to the functioning of all terrestrial ecosystems, owing to their pivotal roles in the decomposition of organic matter and the mineralisation of soil nutrients (Swift et al., 1979). Here, the most widespread saprotroph, recorded in all 29 fellfield soils, was found to be *Pseudogymnoascus roseus*, a fungus that also occurs in both continental Antarctic and high Arctic soils (Zucconi et al., 1996; Bergero et al., 1999; Onofri et al., 2007). Other abundant saprotrophic genera were *Mortierella*, the only representative of the Mucoromycotina recorded here, and a genus that is frequent in cold soils (Onofri et al., 2007; Bridge et al., 2008; Schmidt et al., 2012), along with *Fusarium*, *Pseudeurotium hygrophilum*, *Cladosporium halotolerans*, and *Antarctomyces*, the latter of which was originally described from South Shetland Island soils (Stchigel et al., 2001). The analyses here point to a reduction in the relative abundance of these, and other saprotrophic, fungal taxa as maritime Antarctic soils warm over future decades, with a 3% decrease in the abundance of these fungi in the total community for each degree Celsius rise in MASAT. Given the central role played by saprotrophic fungi in the decomposition of soil organic matter (Waksman, 1931; Lindeberg, 1947; Frankland, 1969), this finding suggests inhibited decay and nutrient cycling in warmer maritime Antarctic soils. However, saprotrophic fungal communities exhibit high levels of functional redundancy, with, for example, a wide range of taxa being capable of simple carbohydrate and cellulose decomposition (Frankland, 1969; Setälä and McLean, 2004). Given a mean of 22 saprotrophic fungal OTUs in each of the soils studied here, it seems unlikely that reductions in the relative abundances of these fungi arising from increases in air temperature of 2–4°C (Bracegirdle et al., 2008; Bracegirdle and Stephenson, 2012) will substantially affect the decomposition process.

Unlike the lichenised and saprotrophic fungal guilds, the yeast growth form, which was dominated by the facultative ascomycetous genera *Capronia*, *Cladophialophora*, and *Rhinocladiella*, and the obligate basidiomycetous genera *Cryptococcus*, *Rhodotorula*, and *Mrakia* – which frequent soils at high latitudes and altitudes (Thomas-Hall et al., 2010; Schmidt et al., 2012; Cox et al., 2016) – was apparently unresponsive to changes in MASAT and the majority of measured edaphic factors. Only soil K concentration and C:N ratio, the latter of which also predicts the composition of yeast communities on other continents (Tedersoo et al., 2014), were significant predictors for the richness and composition of the yeast community. Given the apparent tolerance of the yeast growth form to extreme environments, including both its abundance in soils of the McMurdo Dry Valleys, considered to be some of the most hostile environments for life, and on Mount Howe (87°S), the southernmost mountain on Earth (Atlas et al., 1978; Vishniac, 1996; Fell et al., 2006), increases in yeast abundance in soils at higher latitudes in maritime Antarctica might be expected. However, although such increases are recorded between 69°N and 79°N in the Arctic (Timling et al., 2014), the relative abundance and species richness of yeasts did not vary along the transect studied here.

In contrast to the yeast growth form, the abundance and richness of the lichenised fungal guild varied with latitude. Lichenised fungi accounted for up to 70% of reads from soils at 60–63°S, reflecting the dominance of the symbiosis in all but the wettest habitats of maritime Antarctica (Smith, 2000; Øvstedal and Smith, 2001; Peat et al., 2007), but their abundance and species richness decreased at up to 69°S. This finding contrasts with those of Timling et al. (2014), who showed the frequencies of lichen-forming fungi to increase at higher latitudes in the Arctic. The data here, derived from bare fellfield soils, support the conclusion of Timling et al. (2014) that interactions with higher plants, which are abundant in the Low Arctic and outcompete lichens for light, play an important role in controlling the abundances of lichen-forming fungi in soil. Despite marked decreases in the abundance and richness of lichenised fungi between 60°S and 69°S, subsequent increases in the guild were recorded in soil at Mars Oasis (72°S), suggesting that the oasis, which has a microclimate characterised by midsummer surface soil temperatures of up to 24°C (Convey et al., 2018), may be a favourable habitat not only for soil bacteria and nematodes (Maslen and Convey, 2006; Yergeau et al., 2007b) but also for lichenised fungi as well. Saprotrophic Agaricales similarly declined in relative abundance and richness in soils beyond 63°S, with only two soils at the northern end of the transect generating significant numbers of reads of these taxa. This suggests that the Agaricales, notably species of *Arrhenia*, are poorly adapted to survival in soils at high latitudes, and corroborates field observations that basidiocarps are usually restricted to soils in the South Orkney and South Shetland islands (60–62°S), with very occasional records on the western Antarctic Peninsula to 64–67°S (Pegler et al., 1980; K.K.N., pers. obs.).

In agreement with studies demonstrating that maritime Antarctic soil fungi tend to have bipolar or cosmopolitan distributions (Cox et al., 2016), 16 of the saprotrophic ascomycete genera recorded here were among the 41 genera of fungi found to dominate grassland, shrubland, and forest soils at lower latitudes (Egidi et al., 2019). These fungi were typically free-living saprotrophs with small (length <15 μm; Domsch et al., 2007) conidia, such as *Cladosporium*, *Penicillium*, and *Pseudogymnoascus*, the former of which is capable of intercontinental aerial dispersal to Antarctica (Marshall, 1997). Despite these similarities to soil saprotroph communities at lower latitudes, there are also striking differences between the soil mycoflora of maritime Antarctica and that of other landmasses. Notably, lichenised fungi were abundant in maritime Antarctic fellfield soils, accounting for 19% of all reads in the present study. In contrast, the dominant members of the soil mycoflora on six other continents reported by Egidi et al. (2019) did not include lichenised taxa. While the Ascomycota was the dominant fungal phylum found in the fellfield soils studied here, with 70% of all reads being assigned to the phylum, just 4% of reads were assigned to the Basidiomycota. Conversely, an assessment of fungal diversity in soils under woody plant species in the Americas, Africa, Asia, Australasia, and Europe found that 31 and 56% of all fungi belonged to the Ascomycota and Basidiomycota, respectively, with 50% of all fungal OTUs being assigned to the Agaricomycetes (Tedersoo et al., 2014). As noted elsewhere (Cox et al., 2016; Newsham et al., 2016), the substantial reduction in the abundance and richness of the Basidiomycota in maritime Antarctic soils is most probably owing to the absence of woody plant species, which routinely form ectomycorrhizas with basidiomycetes, and typically Agaricomycetes, at lower latitudes (Smith and Read, 2008).

In addition to *Mortierella*, other basal fungi were recorded here, with the DNA of Glomeromycota occasionally being amplified from fellfield soils, corroborating previous observations that *Deschampsia antarctica* and *Colobanthus quitensis*, the two native Antarctic higher plant species, form sparse arbuscular mycorrhizas in the maritime Antarctic to 62°S (Upson et al., 2008). A single member of the Spizellomycetales (Chytridiomycota) was also recorded at very low frequencies, corroborating the presence of these motile fungi in southern maritime Antarctic and continental Antarctic soils (Bridge and Newsham, 2009; Dreesens et al., 2014), and those at high altitudes receiving significant amounts of snowfall (Schmidt et al., 2012). Other basal phyla or subphyla, typically animal parasites and anaerobic rumen fungi, such as members of the Kickxellomycotina, Zoopagomycotina, Entomophthoromycotina, and Neocallimastigomycotina, were not recorded in Antarctic soil, most probably reflecting an absence of suitable hosts. Nevertheless, it must be noted that the low abundances of these non-Dikaryotic fungi may reflect primer bias, as the gITS7 primer (Ihrmark et al., 2012) has been demonstrated to have mismatches to early diverging fungal lineages (Tedersoo et al., 2015).

## Conclusion

The PERMANOVA and multiple regression analyses reported here indicate significant effects of MASAT on the species richness and relative abundance of the lichenised and saprotrophic fungal guilds, suggesting responses of these assemblages to the warming predicted in maritime Antarctica during the 21st century (Bracegirdle et al., 2008; Bracegirdle and Stephenson, 2012). These analyses also indicate that edaphic factors expected to alter in warmer and less arid maritime Antarctic soils, *viz*., C:N ratio and concentrations of DOC and NH_4_^+^-N, will influence the responses of these guilds to warming. Contrary to previous research in the Arctic (Timling et al., 2014), the analyses here also show that lichenised fungi decrease in abundance at higher latitudes in maritime Antarctica, with reductions in saprotrophic Agaricales also being recorded in more southerly soils. In contrast with vegetated soils on other continents, lichenised fungal taxa were found to be frequent in fellfield soils, reflecting the abundance of the lichen symbiosis in maritime Antarctica. Despite being based on correlative data, the observations here can be used to formulate testable hypotheses in future studies examining the effects of experimental warming on soil fungal diversity in the maritime Antarctic natural environment.

## Data Availability Statement

The datasets presented in this study can be found in the NCBI short read archive (accession code PRJNA282894) and in Dennis et al. (2020) at https://data.bas.ac.uk/full-record.php?id=GB/NERC/BAS/PDC/01402

## Author Contributions

DH, KN, and PD secured funding and conducted fieldwork. PD generated sequence libraries and soil physicochemical data and, along with MD, analyzed data. KN conceived the study and wrote the manuscript, which was commented on and improved by the other authors. All authors contributed to the article and approved the submitted version.

### Conflict of Interest

The authors declare that the research was conducted in the absence of any commercial or financial relationships that could be construed as a potential conflict of interest.
