# Supplementary material for: Regional Diversity of Maritime Antarctic Soil Fungi and Predicted Responses of Guilds and Growth Forms to Climate Change
Source: Front Microbiol. 2021 Jan 26;11:615659. doi: 10.3389/fmicb.2020.615659 (PMC7870798; doi:10.3389/fmicb.2020.615659)
Supplement: Supplementary file 1 [file Table_1.doc]

**Supplementary Table S1**. Summary of the fungal operational taxonomic units (OTUs) recorded across the latitudinal transect. Guilds were assigned using FUNGuild v. 1.0 (Nguyen et al., 2016),

classifying genera as containing lichenized (L) or saprotrophic (S) fungi. The FUNGuild confidence ranking for inclusion was ‘highly probable’ or ‘probable’. Obligate or facultative yeast genera

are marked with asterisks. Genera marked with obelisks are not listed by FUNGuild v. 1.0 as highly probable or probable saprotrophs, but were treated as saprotrophs in analyses owing to ample

evidence of their roles in the decomposition process (Domsch et al., 2007). *Abbreviation*: unc., unclassified.

| **Kingdom** | **Phylum or subphylum** | **Class** | **Order** | **Family** | **Genus** | **Guild** | **No. OTUs** | **Relative abundance (%)** |
| --- | --- | --- | --- | --- | --- | --- | --- | --- |
| Fungi | Ascomycota | Eurotiomycetes | Verrucariales | Verrucariaceae | *Atla* | L | 3 | 0.675 |
|  |  |  |  |  | *Dermatocarpon* | L | 1 | 0.002 |
|  |  |  |  |  | *Heteroplacidium* | L | 1 | 0.002 |
|  |  |  |  |  | *Placopyrenium* | L | 3 | 0.039 |
|  |  |  |  |  | *Polyblastia* | L | 15 | 1.618 |
|  |  |  |  |  | *Sporodictyon* | L | 1 | 0.034 |
|  |  |  |  |  | *Staurothele* | L | 2 | 0.121 |
|  |  |  |  |  | *Thelidium* | L | 2 | 0.022 |
|  |  |  |  |  | *Verrucaria* | L | 54 | 8.544 |
|  |  |  |  |  | *Verruculopsis* | L | 3 | 0.007 |
|  |  |  |  |  | unc. |  | 3 | 1.246 |
|  |  |  | Chaetothyriales | Herpotrichiellaceae | *Capronia** |  | 5 | 0.633 |
|  |  |  |  |  | *Cladophialophora** | S | 14 | 0.429 |
|  |  |  |  |  | *Exophiala** | S | 1 | 0.020 |
|  |  |  |  |  | *Rhinocladiella** | S | 7 | 0.419 |
|  |  |  |  |  | unc. |  | 34 | 1.426 |
|  |  |  |  | unc. | unc. |  | 6 | 0.155 |
|  |  |  | Eurotiales | Trichocomaceae | *Aspergillus* | S | 9 | 0.217 |
|  |  |  |  |  | *Penicillium* | S | 8 | 1.828 |
|  |  |  |  |  | unc. |  | 3 | 0.020 |
|  |  |  | Onygenales | Onygenaceae | unc. |  | 2 | 0.012 |
|  |  |  |  | unc. | unc. |  | 1 | 0.076 |
|  |  |  | Arachnomycetales | Arachnomycetaceae | unc. |  | 1 | 0.207 |
|  |  |  | Mycocaliciales | Mycocaliciaceae | *Mycocalicium* |  | 1 | 0.017 |
|  |  |  | Pyrenulales | Massariaceae | *Massaria* | S | 1 | 0.005 |
|  |  |  | *incertae sedis* | *incertae sedis* | *Sarcinomyces* |  | 1 | 0.002 |
|  |  |  | unc. | unc. | unc. |  | 10 | 0.978 |
|  |  | Lecanoromycetes | Lecanorales | Cladoniaceae | *Cladia* | L | 1 | 0.030 |
|  |  |  |  |  | *Cladonia* | L | 4 | 0.101 |
|  |  |  |  | Lecanoraceae | *Carbonea* |  | 1 | 0.039 |
|  |  |  |  |  | *Lecanora* | L | 6 | 0.108 |
|  |  |  |  |  | *Lecidella* | L | 1 | 0.002 |
|  |  |  |  |  | *Rhizoplaca* | L | 2 | 0.010 |
|  |  |  |  |  | unc. |  | 4 | 0.278 |
|  |  |  |  | Parmeliaceae | *Bryoria* | L | 1 | 0.002 |
|  |  |  |  |  | *Cetrelia* | L | 4 | 0.389 |
|  |  |  |  |  | *Hypotrachyna* | L | 2 | 0.012 |
|  |  |  |  |  | *Montanelia* | L | 1 | 0.214 |
|  |  |  |  |  | *Parmelia* | L | 1 | 0.015 |
|  |  |  |  |  | *Parmelina* | L | 1 | 0.005 |
|  |  |  |  |  | *Pseudephebe* | L | 1 | 0.057 |
|  |  |  |  |  | *Xanthoparmelia* | L | 1 | 0.441 |
|  |  |  |  |  | unc. |  | 3 | 0.020 |
|  |  |  |  | Ramalinaceae | *Bacidina* | L | 1 | 0.012 |
|  |  |  |  |  | unc. |  | 3 | 0.350 |
|  |  |  |  | Stereocaulaceae | *Lepraria* | L | 3 | 0.039 |
|  |  |  |  | Tephromelataceae | *Tephromela* | L | 1 | 0.002 |
|  |  |  |  | *incertae sedis* | *Lecania* | L | 1 | 0.062 |
|  |  |  |  | *incertae sedis* | *Psilolechia* | L | 3 | 0.103 |
|  |  |  |  | *incertae sedis* | unc. |  | 1 | 0.020 |
|  |  |  | Teloschistales | Caliciaceae | *Amandinea* | L | 1 | 0.010 |
|  |  |  |  | Physciaceae | *Buellia* | L | 4 | 0.076 |
|  |  |  |  |  | *Phaeophyscia* | L | 1 | 0.005 |
|  |  |  |  |  | *Physconia* | L | 3 | 0.044 |
|  |  |  |  | Teloschistaceae | *Caloplaca* | L | 11 | 0.337 |
|  |  |  |  |  | *Parvoplaca* | L | 1 | 0.010 |
|  |  |  |  |  | unc. |  | 2 | 0.345 |
|  |  |  | Lecideales | Lecideaceae | *Lecidea* | L | 19 | 1.948 |
|  |  |  | Acarosporales | Acarosporaceae | *Acarospora* | L | 11 | 1.500 |
|  |  |  |  |  | unc. |  | 2 | 0.278 |
|  |  |  | Peltigerales | Lobariaceae | *Lobaria* | L | 2 | 0.732 |
|  |  |  |  |  | *Sticta* | L | 1 | 0.002 |
|  |  |  |  | Pannariaceae | *Psoroma* | L | 6 | 0.185 |
|  |  |  | Pertusariales | Megasporaceae | *Aspicilia* | L | 7 | 0.559 |
|  |  |  | Umbilicariales | Umbilicariaceae | *Omphalodium* | L | 2 | 0.017 |
|  |  |  |  |  | *Umbilicaria* | L | 3 | 0.111 |
|  |  |  | Agyriales | Trapeliaceae | *Placopsis* | L | 5 | 0.534 |
|  |  |  | Ostropales | Stictidaceae | *Cryptodiscus* | S | 1 | 0.047 |
|  |  |  | Rhizocarpales | Catillariaceae | *Austrolecia* | L | 1 | 0.012 |
|  |  | Leotiomycetes | Helotiales | Dermateaceae | *Cryptosporiopsis* | S | 1 | 0.002 |
|  |  |  |  |  | *Pezicula* |  | 1 | 0.005 |
|  |  |  |  |  | unc. |  | 2 | 0.032 |
|  |  |  |  | Helotiaceae | *Claussenomyces* | S | 1 | 0.002 |
|  |  |  |  |  | *Heyderia* | S | 1 | 0.007 |
|  |  |  |  |  | *Hymenoscyphus* |  | 2 | 0.012 |
|  |  |  |  |  | *Rhizoscyphus* |  | 5 | 0.081 |
|  |  |  |  |  | unc. |  | 1 | 0.025 |
|  |  |  |  | Hyaloscyphaceae | *Bryoglossum* | S | 1 | 0.002 |
|  |  |  |  |  | *Hyaloscypha* | S | 3 | 0.219 |
|  |  |  |  |  | *Lachnellula* | S | 1 | 0.002 |
|  |  |  |  |  | unc. |  | 9 | 0.293 |
|  |  |  |  | *incertae sedis* | *Catenulifera* | S | 9 | 0.527 |
|  |  |  |  |  | *Cystodendron* |  | 1 | 0.034 |
|  |  |  |  |  | *Glarea* | S | 1 | 0.202 |
|  |  |  |  |  | *Helicodendron* | S | 1 | 0.012 |
|  |  |  |  |  | *Hyphodiscus* |  | 1 | 0.002 |
|  |  |  |  |  | *Rhexocercosporidium* |  | 1 | 0.002 |
|  |  |  |  |  | *Tetracladium* | S | 12 | 1.071 |
|  |  |  |  |  | unc. |  | 2 | 0.084 |
|  |  |  |  | Rutstroemiaceae | *Scleromitrula* | S | 1 | 0.002 |
|  |  |  |  | unc. | unc. |  | 30 | 1.456 |
|  |  |  | Thelebolales | Thelebolaceae | *Antarctomyces* | S | 12 | 3.941 |
|  |  |  | Leotiales | Leotiaceae | *Alatospora* | S | 6 | 1.131 |
|  |  |  | Erysiphales | Erysiphaceae | *Podosphaera* |  | 1 | 0.017 |
|  |  |  | *incertae sedis* | *incertae sedis* | *Collophora* |  | 1 | 0.005 |
|  |  |  |  |  | *Geomyces* | S | 4 | 0.034 |
|  |  |  |  |  | *Leohumicola* | S | 1 | 0.002 |
|  |  |  | unc. | unc. | unc. |  | 5 | 0.204 |
|  |  | Dothideomycetes | Capnodiales | Davidiellaceae | *Cladosporium*^†^ | S | 6 | 1.584 |
|  |  |  |  | *incertae sedis* | *Capnobotryella* | S | 2 | 0.005 |
|  |  |  |  | *incertae sedis* | *Friedmanniomyces* |  | 1 | 0.015 |
|  |  |  |  | *incertae sedis* | *Toxicocladosporium* |  | 2 | 0.153 |
|  |  |  |  | Mycosphaerellaceae | *Microcyclus* |  | 4 | 0.091 |
|  |  |  |  |  | *Mycosphaerella* |  | 1 | 0.034 |
|  |  |  |  |  | *Pseudocercospora* |  | 1 | 0.002 |
|  |  |  |  | Teratosphaeriaceae | *Teratosphaeria* |  | 1 | 0.187 |
|  |  |  |  | unc. | unc. |  | 2 | 0.010 |
|  |  |  | Pleosporales | *incertae sedis* | *Ascochyta* |  | 1 | 0.007 |
|  |  |  |  | *incertae sedis* | *Peyronellaea* | S | 1 | 0.010 |
|  |  |  |  | *incertae sedis* | *Phoma*^†^ | S | 2 | 0.131 |
|  |  |  |  | Phaeosphaeriaceae | *Phaeosphaeria* | S | 1 | 0.022 |
|  |  |  |  |  | *Stagonospora* |  | 1 | 0.022 |
|  |  |  |  |  | unc. |  | 1 | 0.005 |
|  |  |  |  | Pleomassariaceae | *Prosthemium* | S | 1 | 0.002 |
|  |  |  |  | Pleosporaceae | *Bipolaris* |  | 1 | 0.002 |
|  |  |  |  |  | *Cochliobolus* |  | 1 | 0.054 |
|  |  |  |  | Sporormiaceae | *Preussia* | S | 1 | 0.012 |
|  |  |  |  | unc. | unc. |  | 5 | 0.335 |
|  |  |  | Botryosphaeriales | Botryosphaeriaceae | *Neofusicoccum* |  | 2 | 0.015 |
|  |  |  | Dothideales | Dothioraceae | *Aureobasidium*,*^†^ | S | 1 | 0.012 |
|  |  |  | Patellariales | Patellariaceae | *Rhytidhysteron* | S | 1 | 0.005 |
|  |  |  | *incertae sedis* | *incertae sedis* | *Cryomyces* |  | 2 | 0.020 |
|  |  |  | *incertae sedis* | Myxotrichaceae | *Gymnostellatospora* | S | 5 | 0.025 |
|  |  |  |  |  | *Myxotrichum* | S | 1 | 0.005 |
|  |  |  |  |  | *Oidiodendron* |  | 1 | 0.015 |
|  |  |  |  |  | unc. |  | 1 | 0.002 |
|  |  |  | *incertae sedis* | Pseudeurotiaceae | *Pseudeurotium* | S | 15 | 4.655 |
|  |  |  |  |  | *Pseudogymnoascus* | S | 15 | 5.079 |
|  |  |  | unc. | unc. | unc. |  | 5 | 0.084 |
|  |  | Sordariomycetes | Hypocreales | Clavicipitaceae | *Claviceps* |  | 1 | 0.002 |
|  |  |  |  |  | *Drechmeria* |  | 4 | 0.052 |
|  |  |  |  |  | *Pochonia* |  | 3 | 0.034 |
|  |  |  |  | Cordycipitaceae | *Lecanicillium* |  | 1 | 0.022 |
|  |  |  |  |  | *Simplicillium* |  | 1 | 0.002 |
|  |  |  |  | Hypocreaceae | *Acrostalagmus* |  | 1 | 0.027 |
|  |  |  |  | *incertae sedis* | *Acremonium*^†^ | S | 2 | 0.207 |
|  |  |  |  |  | *Ilyonectria* |  | 1 | 0.039 |
|  |  |  |  | Nectriaceae | *Fusarium*^†^ | S | 7 | 6.438 |
|  |  |  |  |  | unc. |  | 1 | 0.259 |
|  |  |  |  | Ophiocordycipitaceae | *Chaunopycnis* |  | 1 | 0.002 |
|  |  |  |  |  | *Tolypocladium* |  | 4 | 0.089 |
|  |  |  |  |  | unc. |  | 1 | 0.076 |
|  |  |  |  | unc. | unc. |  | 4 | 0.145 |
|  |  |  | Sordariales | Chaetomiaceae | *Humicola* | S | 1 | 0.054 |
|  |  |  |  | Lasiosphaeriaceae | *Podospora* |  | 1 | 0.032 |
|  |  |  | *incertae sedis* | Magnaporthaceae | unc. |  | 1 | 0.027 |
|  |  |  |  | Plectosphaerellaceae | *Musicillium* |  | 1 | 0.007 |
|  |  |  | unc. | unc. | unc. |  | 8 | 0.461 |
|  |  | Pezizomycetes | Pezizales | Pezizaceae | *Peziza*^†^ | S | 1 | 0.108 |
|  |  |  |  | Pyronemataceae | *Cheilymenia* | S | 1 | 0.005 |
|  |  |  | unc. | unc. | unc. |  | 4 | 0.195 |
|  |  | Orbiliomycetes | Orbiliales | Orbiliaceae | unc. |  | 2 | 0.027 |
|  |  | Saccharomycetes | Saccharomycetales | *incertae sedis* | *Debaryomyces** |  | 1 | 0.020 |
|  |  | *incertae sedis* | *incertae sedis* | *incertae sedis* | *Chalara* |  | 2 | 0.089 |
|  |  |  |  |  | *Cladochasiella* |  | 1 | 0.002 |
|  |  |  |  |  | *Seifertia* |  | 1 | 0.441 |
|  |  | unc. | unc. | unc. | unc. |  | 50 | 11.187 |
|  |  |  |  |  |  |  |  |  |
|  | Basidiomycota | Tremellomycetes | Cystofilobasidiales | Cystofilobasidiaceae | *Guehomyces** | S | 1 | 0.002 |
|  |  |  |  |  | *Mrakia** | S | 5 | 0.227 |
|  |  |  | Filobasidiales | Filobasidiaceae | *Cryptococcus** |  | 3 | 0.300 |
|  |  |  |  |  | *Filobasidium** | S | 1 | 0.010 |
|  |  |  | Tremellales | *incertae sedis* | *Dioszegia** |  | 2 | 0.007 |
|  |  |  |  | *incertae sedis* | *Tremella** |  | 2 | 0.020 |
|  |  |  |  |  | unc. |  | 1 | 0.022 |
|  |  |  |  | unc. | unc. |  | 2 | 0.007 |
|  |  |  | unc. | unc. | unc. |  | 5 | 0.271 |
|  |  | Microbotryomycetes | *incertae sedis* | *incertae sedis* | *Glaciozyma** |  | 1 | 0.010 |
|  |  |  | Leucosporidiales | Leucosporidiaceae | *Leucosporidiella** | S | 3 | 0.165 |
|  |  |  |  |  | *Mastigobasidium** | S | 3 | 0.039 |
|  |  |  | Sporidiobolales | *incertae sedis* | *Rhodotorula** | S | 8 | 0.635 |
|  |  |  |  | *incertae sedis* | *Sporobolomyces** | S | 1 | 0.005 |
|  |  |  |  | unc. | unc. |  | 4 | 0.200 |
|  |  |  | unc. | unc. | unc. |  | 4 | 0.564 |
|  |  | Agaricomycetes | Agaricales | Agaricaceae | *Coprinus* | S | 1 | 0.002 |
|  |  |  |  |  | *Lycoperdon* | S | 1 | 0.002 |
|  |  |  |  | Psathyrellaceae | unc. |  | 1 | 0.002 |
|  |  |  |  | Tricholomataceae | *Arrhenia* | S | 6 | 0.362 |
|  |  |  |  | unc. | unc. |  | 1 | 0.002 |
|  |  |  | Cantharellales | Ceratobasidiaceae | *Ceratobasidium* |  | 1 | 0.002 |
|  |  |  | Hymenochaetales | Hymenochaetaceae | unc. |  | 1 | 0.025 |
|  |  |  |  | Schizoporaceae | *Hyphodontia* | S | 1 | 0.027 |
|  |  |  | Sebacinales | Sebacinaceae | unc. |  | 1 | 0.005 |
|  |  |  |  | unc. | unc. |  | 1 | 0.017 |
|  |  |  | unc. | unc. | unc. |  | 1 | 0.020 |
|  |  | Agaricostilbomycetes | Agaricostilbales | Agaricostilbaceae | *Sterigmatomyces** |  | 1 | 0.005 |
|  |  | Exobasidiomycetes | *incertae sedis* | *incertae sedis* | *Tilletiopsis** |  | 1 | 0.022 |
|  |  | Pucciniomycetes | Platygloeales | Eocronartiaceae | *Eocronartium* |  | 1 | 0.007 |
|  |  | Ustilaginomycetes | Ustilaginales | unc. | unc. |  | 1 | 0.015 |
|  |  | Wallemiomycetes | Wallemiales | Wallemiaceae | *Wallemia* | S | 1 | 0.002 |
|  |  | *incertae sedis* | Malasseziales | *incertae sedis* | *Malassezia** |  | 5 | 0.079 |
|  |  | unc. | unc. | unc. | unc. |  | 8 | 1.276 |
|  |  |  |  |  |  |  |  |  |
|  | Mucoromycotina | *incertae sedis* | Mortierellales | Mortierellaceae | *Mortierella*^†^ | S | 46 | 8.806 |
|  |  |  |  | unc. | unc. |  | 3 | 0.057 |
|  |  |  |  |  |  |  |  |  |
|  | Glomeromycota | Glomeromycetes | Glomerales | Glomeraceae | unc. |  | 1 | 0.002 |
|  |  | unc. | unc. | unc. | unc. |  | 1 | 0.005 |
|  |  |  |  |  |  |  |  |  |
|  | Chytridiomycota | Chytridiomycetes | Spizellomycetales | Spizellomycetaceae | *Triparticalcar* |  | 1 | 0.002 |
|  |  |  |  |  |  |  |  |  |
|  | unc. | unc. | unc. | unc. | unc. |  | 52 | 3.897 |
|  |  |  |  |  |  |  |  |  |
| No BLAST hit |  |  |  |  |  |  | 399 | 13.700 |
