# Supplementary figures and images for: Regional Diversity of Maritime Antarctic Soil Fungi and Predicted Responses of Guilds and Growth Forms to Climate Change
Source: Front Microbiol. 2021 Jan 26;11:615659. doi: 10.3389/fmicb.2020.615659 (PMC7870798; doi:10.3389/fmicb.2020.615659)

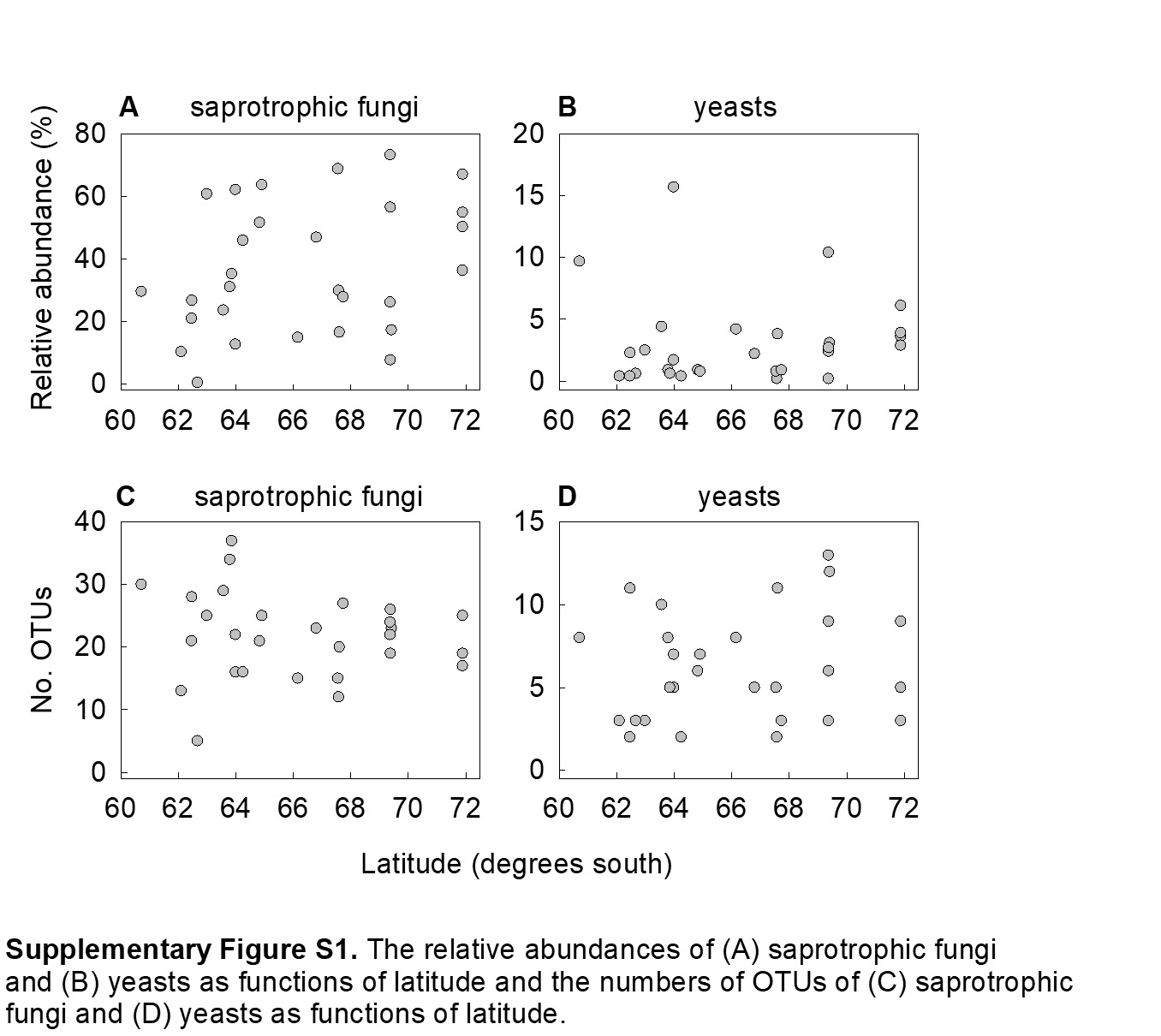

Supplement: Supplementary file 2 [file Image_1.jpeg]
